# Gegen Qinlian Decoction Treats Diarrhea in Piglets by Modulating Gut Microbiota and Short-Chain Fatty Acids

**DOI:** 10.3389/fmicb.2019.00825

**Published:** 2019-04-18

**Authors:** Chang-Shun Liu, Xiao Liang, Xiao-Han Wei, Zhen Jin, Fei-Long Chen, Qing-Fa Tang, Xiao-Mei Tan

**Affiliations:** ^1^School of Traditional Chinese Medicine, Southern Medical University, Guangzhou, China; ^2^Guangdong Provincial Key Laboratory of Chinese Medicine Pharmaceutics, Southern Medical University, Guangzhou, China; ^3^Guangdong Provincial Engineering Laboratory of Chinese Medicine Preparation Technology, Guangzhou, China

**Keywords:** gut microbiota, short-chain fatty acids, Gegen Qinlian decoction, diarrhea, anti-inflammation

## Abstract

Gut microbiota and its metabolites, short-chain fatty acids (SCFAs), play important roles in diarrheal diseases. Gegen Qinlian decoction (GQD), a Chinese herb formula, has been widely used to treat infectious diarrhea for centuries. However, little is known about the mechanism underlying its efficacy and whether it is mediated by gut microbiota and SCFAs. In this study, the composition of gut microbiota from bacterial diarrheal piglets was assessed using 16S rRNA analysis. The concentrations of fecal SCFAs were determined using a gas chromatography-mass spectrometer (GC-MS). The expression of mucosal pro-inflammatory cytokines in the colon was ascertained. Results showed that GQD reverses the reduction in the richness of gut microbiota, changes its structure, and significantly increases the relative abundances of SCFA-producing bacteria, including *Akkermansia, Bacteroides, Clostridium, Ruminococcus*, and *Phascolarctobacterium*. Moreover, GQD increased the levels of fecal SCFAs, including acetic acid, propionic acid, and butyric acid. GQD thus attenuates diarrhea in piglets. Further, our results suggest that the SCFAs could help to attenuate mucosal pro-inflammatory responses following GQD treatment by inhibiting histone deacetylase and the NF-κB pathway. We thus suggseted that gut microbiota play an important role during diarrhea treatment, an effect may be promoted by the GQD-induced structural changes of the gut microbial community and production of SCFAs. The increased levels of SCFAs probably provide further help to attenuate mucosal inflammation and diarrhea. In conclusion, our study might provide evidence that GQD treats diarrhea maybe involved in modulating gut microbiota and increasing SCFA levels.

## Introduction

Diarrheal diseases cause approximately 1.7 billion new infections and 0.76 million deaths in children of under 5 years of age annually and are thus the second most common cause of mortality in young children in developing countries (Kotloff et al., 2012, 2013). Outbreaks of such diseases are usually related to infection by common causative agents, mainly rotavirus, *Salmonella enterica*, and diarrheagenic *Escherichia coli* (Hodges and Gill, 2010; Kolenda et al., 2015).

Recent studies have suggested that diarrhea is strongly related to the dysbiosis of gut microbiota (Shrivastava et al., 2017; The et al., 2017). Gut microbiota mainly colonize the large intestine. Their capacity to help colonic fermentation, stimulation of the immune system, and colonization resistance against diarrheal pathogens underlie their importance to human health (Baumler and Sperandio, 2016; Zhang et al., 2017). However, childhood colonization resistance of gut microbiota is weak due to the immaturity of intestinal micro-ecology. The perturbations of the gut microbial community by various etiological agents is one of the main pathogeneses during the early phase of diarrheal infection (Vogt and Finlay, 2017); pathogens could induce diarrhea by transient reversals of the enteric levels of *Bacteroides* and *Prevotella* (David et al., 2015). Prior research has also found that a consistent elevation of *Escherichia* and *Fusobacterium* and a significant depletion of *Bifidobacterium* were observed in diarrheal children relative to healthy children (Shrivastava et al., 2017; The et al., 2017). Therefore, modulation of the gut microbial community can prevent and treat diarrhea by promoting competition for nutrients and producing metabolites that inhibit pathogen growth or virulence, thereby increasing colonization resistance against diarrheal pathogens.

Gut microbiota metabolites short-chain fatty acids (SCFAs), defined as 1–6 carbon volatile fatty acids, are key signaling molecules between the gut microbiota and the host. SCFAs provide energy to the host cells and gut microbiota, shape the gut environment, and regulate the immune system, which subsequently regulates the intestinal physiology (Ríos-Covián et al., 2016). The disturbance of gut microbiota by diarrheal diseases is partly accounted for by the changes in SCFA levels, especially those of butyric acid and propionic acid. These changes consequently weaken the immunological and regulatory functions of SCFAs. Investigations have demonstrated that fecal SCFAs could serve as a valid, reliable diagnostic biomarker for diarrheal diseases (Treem et al., 1996; Farup et al., 2016). Moreover, as dietary regulation of SCFA levels stimulates intestinal water and ion absorption and reduces intestinal permeability and mucosal inflammation (Sivaprakasam et al., 2017; van der Beek et al., 2017), promoting SCFAs could alleviate diarrhea and maintain intestinal homeostasis (Krokowicz et al., 2014).

Gegen Qinlian decoction (GQD), a Chinese herbal formula, has been widely used for centuries to treat gastrointestinal diseases, especially infectious diarrhea (Xu B. et al., 2015). However, the mechanism underlying the effect of GQD on diarrhea remains unclear. Interestingly, it has also been demonstrated that GQD treatment could alleviate type 2 diabetes by modulating gut microbiota and increasing the abundance of probiotics and SCFA-producing bacteria, such as *Bifidobacterium*, *Faecalibacterium*, and *Butyricimonas* (Xu J. et al., 2015). The efficacy of GQD in the treatment of steatohepatitis is also related to the modulation of gut microbiota (Guo et al., 2018). Moreover, prior research has shown that some components of GQD, including berberine and baicalin, may regulate the structure of gut microbiota (Han et al., 2017; Cui et al., 2018). Based on these observations, we hypothesized that GQD could modulate the structure of gut microbiota and SCFA levels. Specifically, we explored whether GQD could regulate the structure of gut microbiota in diarrhea. We further studied the influence of GQD on the SCFA levels, as well as the effect of the latter on mucosal inflammatory responses in the colon. The present study used piglets to investigate the antidiarrheal efficacy of GQD due to their anatomical, physiological, and intestinal microbial-composition similarities with human beings (Heinritz et al., 2013).

## Materials and Methods

### Chemicals and Biological Materials

Reference standards were purchased from the National Institute for the Control of Pharmaceutical and Biological Products (Beijing, China), including acetic acid, propionic acid, butyric acid, *iso*-butyric acid, valeric acid, *iso*-valeric acid, and 2-ethylbutyric acid (internal standard, IS; purity of >98.0%). Sulfuric acid, ether, and other chemicals adhered to analytical grade.

### Preparation of GQD

The GQD formula includes *Pueraria lobata* (Wild.) Ohwi (root, Leguminosae), *Scutellaria baicalensis* Georgi (rhizome, Lamiaceae), *Coptis chinensis* Franch (rhizome, Ranunculaceae), and *Glycyrrhiza uralensis* Fisch (rhizome, Leguminosae). All the herbs were purchased from the Kangmei Pharmaceutical Co., Ltd. (Guangzhou, China). GQD was prepared according to the method of the Chinese Pharmacopoeia (Chinese Pharmacopoeia Commission, 2015). The herbal material *P. lobata* (Wild). Ohwi (15 g), was soaked in 400 mL of cold water for 30 min before being boiled for 20 min alone. The other herbs, including *S. baicalensis* Georgi (9 g), *C. chinensis* Franch. (9 g), and *G. uralensis* Fisch. (6 g), were added and boiled together with *P. lobata* (Wild.) Ohwi for 10 min. The first decoction was thus obtained. The mixture was boiled a second time with an addition of 300 mL water for 30 min to obtain the second decoction. Finally, the first and second decoctions were mixed, filtered through gauze, and concentrated to a final volume of 130 mL. GQD samples were analyzed using a high-performance liquid chromatography (Supplementary Figure 1).

### Animal Experiments

#### Animals

Tibetan miniature pigs (age, 20 ± 2 d; weight, 2.0 ± 0.5 kg; 50% female) were provided by the Experimental Animal Center, Southern Medical University (Guangzhou, China). Piglets were acclimatized to the laboratory for 3 days prior to the experiments. Animals were housed in strictly controlled conditions: a temperature of 25 ± 1°C, relative humidity of 65 ± 10%, and 12-h light-dark cycles. All studies were performed in accordance with the proposals of the Committee for Research and Ethical Issues of the International Association for the Study of Pain and were approved by the Animal Ethics Committee of Southern Medical University (Approval number: L2018026).

#### Experimental Processes

The animal experiments were conducted by referencing the methods of a similar study with minor modifications (Heinritz et al., 2016). We randomly divided the 12 piglets into normal control (NC), model control (MC), and GQD groups (*n* = 4). The NC group was treated with water, while the MC and GQD animals were orally administered *E. coli* (O8:K91, K88ac; China Veterinary Culture Center, Beijing, China) at a dosage of 10^9^ CFU/kg. The bacterial diarrheal model was successfully implemented when piglets maintained watery diarrhea for more than 6 h. The GQD group was then treated with orally administered GQD for a week, while the NC and MC groups were treated with water. Feces samples defecated in 0.5 h were collected in sealed sterile plastic tubes every day. Feces were frozen immediately with liquid nitrogen after sampling and stored at -80°C until the DNA extraction. Colon tissue was collected at 7 days after the animals were sacrificed and were immediately stored at -80°C.

### Analysis of Fecal 16S rRNA

The composition of gut microbiota was detected by 16S rRNA sequencing analysis. Microbial genomic DNA was extracted from fecal samples using a MoBio Fecal DNA extraction kit (MoBio Laboratories Inc., Carlsbad, CA, United States) according to the manufacturer’s protocols. The extracted DNA from each fecal sample was amplified at the V4 region of the 16S rRNA genes by using specific primers: 515F (5′-GTGCCAGCMGCCGCGGTAA-3′) and 806R (5′-GGACTACHVGGGTWTCTAAT-3′). Amplicons were extracted from 1% agarose gels, purified by Agencourt AMPure XP (Beckman Coulter, Fullerton, CA, United States), and quantified via real-time quantitative PCR. The qualified amplicons were pooled into equimolar fractions and paired-end sequenced (PE250) on an Illumina MiSeq platform (Beijing Genomics Institute, Shenzhen, China). Reads were assembled using FLASH (version 1.2.11) and analyzed using QIIME (MacQIIME version 1.8.0). After quality filtering and chimera removal, clean sequences in each sample were clustered into operational taxonomic units (OTUs) with 97% nucleotide identity using UPARSE (version 7.1). The phylogenetic affiliation of each 16S rRNA gene sequence was assigned by the RDP classifier.

### Determination of Fecal SCFA

#### Instrumental Condition

The concentrations of fecal SCFAs was determined using a gas chromatography-mass spectrometer (GC-MS) system. The GC-MS analysis was performed using a TRACE1300 gas chromatograph equipped with an ISQ Single Quadrupole Mass Spectrometer and AI1300 auto-sampler (Thermo Scientific). A high polarity capillary column with polyethylene glycol phase coating (TG-WAXMS, Capillary GC Column; 30 m × 0.25 mm ID, coated with 0.25 μm film thickness; Thermo Fisher Scientific Inc., MA, United States) was used to separate the SCFAs. A volume of 1 μL of aliquot sample was automatically injected into the inlet, which was maintained at a temperature of 200°C with a 1:5 split mode. Nitrogen (purity of 99.999%) was used as a carrier gas with a flow rate of 1 mL/min. The oven program was adopted at an initial temperature of 90°C for 1 min, increased to 200°C at a rate of 12°C/min, and maintained at 200°C for 2.33 min. The selected ion mode was chosen to determine the ion mass of each SCFA, including 43, 45, 57, 60, 73, 74, and 87. The temperatures of the ion source and injection port were 250 and 230°C, respectively. The flow rates of hydrogen, air, and nitrogen as composition gasses were 30, 300, and 30 mL/min, respectively. The runtime for each analysis was 12.5 min. Data was handled in the Thermo Xcalibur Qual Browser.

#### Sample Preparation

Feces (0.5 g) were weighed. Water (0.5 mL) was then added, and the combination was mixed ultrasonically for 20 min. The mixture was then centrifuged at 20,385 g and 4°C for 10 min. The supernatant (0.5 mL) was removed and collected into an EP tube containing 0.3 g of anhydrous sodium sulfate. We then added 50% sulfate acid (10 μL), and ether (1 mL) and vortex-mixed the solution. The supernatant was obtained by centrifugation (2265 g and 4°C for 10 min). Finally, the supernatant (800 μL) and IS solution (0.86 μL) were blended and transferred to autosampler vials for determination of the SCFAs by GC-MS.

### Immunohistochemistry

Colon tissue was fixed in 4% neutral buffered formalin for 24 h. The tissue was embedded in paraffin and sliced to a thickness of 4 μm using a microtome (Leica, Nussloch, Germany). For the evaluation of colon mucosa, hematoxylin and eosin Y (HE) stains were used on the prepared slides. Images of each slide at 400 × magnification were obtained using a light microscope (Olympus, Tokyo, Japan). The histological score was used for the evaluation of slices based on the severity of inflammation and enterocyte arrangement (Supplementary Table 1).

To examine the expression of cytokines, slices were incubated with anti-pig-ZO-1, anti-Occludin, anti-TNF-α, anti-IL-6, anti-histone deacetylase1 (HDAC1), anti-histone deacetylase2 (HDAC2), anti-IκBα, and anti-NF-κB P65 (Abcam, Cambridge, MA, United States) antibodies at 25°C for 1 h, followed by incubation with secondary antibodies at 37°C for 20 min. After washing with phosphate-buffer saline, staining was coupled with 3, 3′-diaminobenzidine for color development. All slices were counterstained with hematoxylin at 25°C for 1 min. Microscopic images at 400 × magnification of each slide were obtained with a light microscope.

### ELISA Test for TNF-α, IL-6, and HDAC and NF-κB P65 Binding Activities

The frozen colon tissue was weighed and then homogenized on the ice surface. Homogenate was obtained by centrifugation at 1000 *g* and 4°C for 20 min. TNF-α and IL-6 levels of colonic homogenate were determined using ELISA kits (Beijing Cheng Lin Biological Technology Co., LTD., Beijing, China) according to the manufacturer’s protocol. The HDAC activity in the nuclear extract of colon tissue was measured using the Epigenase HDAC Activity/Inhibition Direct Assay Kit (Epigentek Group Inc., United States) and following the manufacturer’s instructions. Moreover, the binding capacity of nuclear P65 to the NF-κB consensus site in the nuclear extract of colon tissue was evaluated by measuring the activated NF-κB P65 using the ELISA-based *Trans*-AM^TM^ NF-κB P65 Kit (Active Motif, Carlsbad, United States); this evaluation was performed according to the manufacturer’ instructions by referencing the similar study (Cichocki et al., 2014). The results were expressed as absorbance (OD_450__nm_/mg protein).

### Western Blot Analysis for HDAC1, HDAC2, IκBα, and NF-κB p65

Total protein was extracted from colon tissues using a radioimmunoprecipitation assay buffer (Beyotime, Shanghai, China) and quantified using a BCA protein assay kit (Bio-Rad, Hercules, CA, United States). Equal total protein (45 μg) was loaded and separated using 10% sodium dodecyl sulfate polyacrylamide gel electrophoresis and then transferred to a polyvinylidene difluoride membrane. The membranes were blocked in 5% non-fat milk and incubated with primary antibodies overnight at 4°C. Subsequently, the membranes were incubated with secondary antibodies for 1 h at 25°C. After incubation with the primary and secondary antibodies, the filters were washed thrice in TBST. Protein bands were developed and quantified through densitometry analysis using an Alpha Innotech imaging system (San Leandro, CA, United States). The results were normalized to those for β-actin.

### Data Analysis

Each value was presented as a mean ± standard deviation and analyzed via SPSS 20.0 software. Significant differences between groups were assessed by the student’s *t*-test analysis, analysis of variance, or Kruskal–Wallis test. Differences were considered statistically significant at *p* < 0.05.

## Results

### Efficacy of GQD on Alleviating Diarrhea

**Figure 1:**
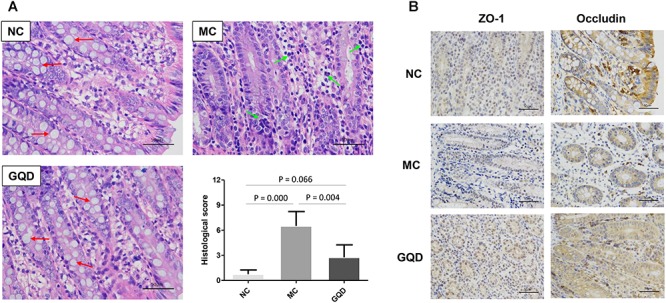
GQD alleviated intestinal mucosal injury in diarrheal piglets after treatment. **(A)** Status of colonic mucosa. Red arrows present the goblet cells and green arrows present the infiltrating cells. **(B)** Expression of the tight junction proteins (ZO-1 and Occludin).

The antidiarrheal efficacy of GQD was informed by the conditions of feces and colonic mucosal injury. GQD treatment alleviated diarrheal symptoms by 3 days (Supplementary Figure 2) and significantly decreased the fecal water content of diarrheal piglets (*P* < 0.05, Table 1). Moreover, HE staining revealed that the MC group featured more infiltrating cells, fewer goblet cells, and looser intercellular space compared to the NC and GQD groups. The histological score of the GQD group was remarkably reduced compared to that of the MC group (*P* < 0.01, Figure 1A). The expressions of tight junction proteins (ZO-1 and occludin) were down-regulated in the diarrheal condition, which indicated an increase in gut permeability. However, GQD reversed these changes (Figure 1B).

**Table 1:** Water contents of feces during the GQD treatment.

Water contents (%) (days)	NC	MC	GQD
0	69.50 ± 3.62	90.63 ± 0.53**	91.48 ± 0.91**
1	65.08 ± 1.16	89.82 ± 3.30**	71.68 ± 0.52^∗^††
3	65.07 ± 1.55	75.48 ± 4.97**	71.73 ± 0.22^∗^†
5	64.49 ± 5.90	74.15 ± 3.00*	66.87 ± 3.46††
7	65.47 ± 0.51	69.21 ± 5.47*	64.35 ± 4.70†

^∗^*P < 0.05, ^∗∗^*P* < 0.01, vs. NC; †*P* < 0.05, ††*P* < 0.01, vs. MC*.

### Overall Structural Modulation of Gut Microbiome During GQD Treatment

**Figure 2:**
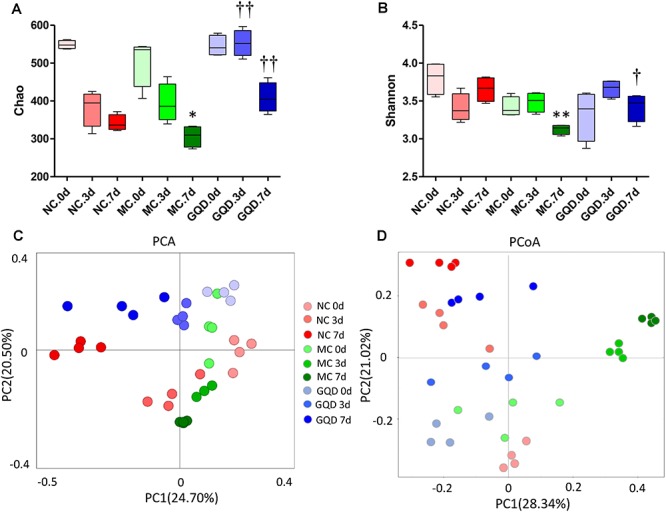
GQD changed the structure of gut microbiota in diarrheal piglets. **(A)** Chao and **(B)** Shannon indexes calculated after rarefying to an equal number of sequence reads for all samples, GQD improved the Chao and Shannon indexes compared with the MC group; **(C)** PCA analysis suggested the structural moderation of gut microbiota during the treatment. **(D)** PCoA score based on weighted Unifrac metrics indicated the different beta diversity of gut microbiota between GQD and MC groups. ^∗^*P* < 0.05, ^∗∗^*P* < 0.01, vs. NC; †*P* < 0.05, ††*P* < 0.01, vs. MC.

16S-rRNA sequencing analysis was used to investigate the structural changes in the gut microbiota in piglets that received GQD treatment. In total, 3,771,781 useable reads were obtained from 36 samples, with an average of 104,771 ± 909 reads per sample, and 978 OTUs were collected. After treatment, the Chao and Shannon indexes were higher in the GQD and NC groups compared to the MC group (*P* < 0.05, Figure 2A,B). The principal component analysis (PCA) and principal coordinates analysis (PCoA) revealed that the structures of gut microbiota differed between the diarrheal and healthy piglets (Supplementary Figure 3). However, after administration of GQD, gut microbiota in the GQD group gradually varied from the MC group over the course of the experiment. By 7 days, the distance between the GQD and NC groups were significantly closer than that between the GQD and MC groups (Figure 2C,D). These results suggest that the gut microbiota structure changed significantly in response to GQD administration.

### Key Phylotypes of Gut Microbiome Modulated by GQD Treatment

**Figure 3:**
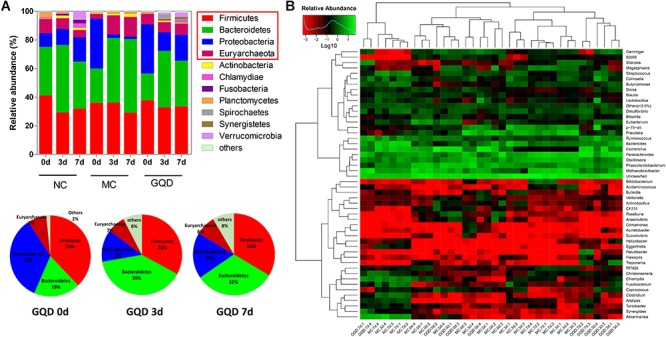
Relative abundance of most abundant OTUs in fecal samples during the treatment. **(A)** Relative abundance of most abundant OTUs at the phylum level in each group. Pie charts showed the recovery of gut microbial composition in the GQD group over the course of treatment. **(B)** Heatmap of most abundant OTUs at the genus level of each sample. The color of spots in the panel represents the mean relative abundance (normalized and log10-transformed) of the OTU in each group. The OTUs are organized basing on their phylogenetic positions.

The taxon-based analysis showed significant changes in the gut microbial composition in response to GQD treatment. Moreover, marked differences at both the phylum and the genus levels were observed among NC, MC, and GQD samples (Figure 3). Eleven phyla could be found in all samples, the most abundant of which were *Firmicutes, Bacteroidetes, Proteobacteria*, and *Euryarchaeota*. Compared to the NC group, the quantities of *Proteobacteria* and *Bacteroidetes* were significantly increased and reduced, respectively, by diarrhea (0 days). However, GQD reversed these changes after treatment (7 days) (*P* < 0.01, Figure 3A).

Twelve of 46 genera were significantly different after treatment, as determined by the Kruskal–Wallis test (*P* < 0.05, Table 2 and Figure 3B). Within the NC group, Bacteroides (17.26%), Escherichia (13.10%), Clostridium (7.74%), and Akkermansia (6.80%) were the most abundant. These abundances differed significantly from those observed in the MC group: Bacteroides (2.2%), Escherichia (0.15%), Clostridium (0.34%), Akkermansia (0.01%), Methanobrevibacter (13.68%), and Oscillospira (10.00%). In the GQD group, the enriching effects on Bacteroides (3.17%), Escherichia (10.92%), Clostridium (2.39%), and Akkermansia (3.87%) and inhibitory effects on Methanobrevibacter (7.93%) and Oscillospira (3.79%) were observed relative to the abundances observed in the MC group (*P* < 0.05). Further, at the species level, the quantities of *Akkermansia muciniphila*, *Bacteroides uniformis*, *B. fragilis*, *Clostridium citroniae*, *C. symbiosum*, *C. hathewayi*, and *Ruminococcus torques* decreased in the MC group compared to the other groups. The levels of these bacteria increased significantly after GQD treatment (*P* < 0.05; Table 3).

**Table 2:** Relative abundance of bacteria at the genus level in each group after treatment.

Relative abundance (%)	NC	MC	GQD
Akkermansia	6.80 ± 2.22	0.01 ± 0.00**	3.87 ± 4.47**††
Bacteroides	17.26 ± 2.69	2.21 ± 0.71**	3.17 ± 1.31**†
Butyricimonas	2.77 ± 0.60	0.69 ± 0.07**	0.85 ± 0.30**†
Clostridium	7.74 ± 1.00	0.34 ± 0.05**	2.39 ± 1.31**††
Ruminococcus	4.73 ± 1.52	0.65 ± 0.19**	6.71 ± 3.87††
Phascolarctobacterium	2.98 ± 0.60	2.09 ± 0.27*	4.45 ± 3.35†
Escherichia	13.10 ± 3.17	0.15 ± 0.05**	10.92 ± 9.46††
Streptococcus	3.50 ± 1.09	0.18 ± 0.03**	0.35 ± 0.04**†
Desulfovibrio	1.98 ± 0.26	0.78 ± 0.38**	1.72 ± 0.13††
Methanobrevibacter	5.02 ± 0.59	13.68 ± 1.82**	7.93 ± 2.42**††
Oscillospira	3.70 ± 2.22	10.00 ± 1.56**	3.79 ± 1.24††
Prevotella	0.51 ± 0.43	4.79 ± 1.66**	0.30 ± 0.13††

^∗^*P < 0.05, ^∗∗^*P* < 0.01, vs. NC; †*P* < 0.05, ††*P* < 0.01, vs. MC*.

**Table 3:** Relative abundance of bacteria at the species level in each group after treatment.

Relative abundance (%)	NC	MC	GQD
Akkermansia muciniphila	5.78 ± 0.65	0.00 ± 0.00**	2.46 ± 1.27**††
Bacteroides uniformis	6.17 ± 0.71	1.03 ± 0.21**	2.28 ± 0.21**††
Bacteroides fragilis	0.73 ± 0.27	0.16 ± 0.02**	0.40 ± 0.15^∗^†
Bacteroides ovatus	0.41 ± 0.05	0.06 ± 0.01**	0.51 ± 0.20††
Clostridium citroniae	2.47 ± 0.42	0.05 ± 0.02**	0.24 ± 0.01**††
Clostridium symbiosum	1.31 ± 0.57	0.12 ± 0.00**	0.53 ± 0.00**††
Clostridium hathewayi	0.91 ± 0.07	0.03 ± 0.00 **	0.18 ± 0.06 **††
Clostridium lavalense	0.39 ± 0.03	0.02 ± 0.01**	0.17 ± 0.13††
Ruminococcus torques	1.15 ± 0.48	0.00 ± 0.00**	0.20 ± 0.09**††
Collinsella aerofaciens	1.25 ± 0.79	0.19 ± 0.09**	0.61 ± 0.29†
Escherichia coli	12.36 ± 3.58	0.15 ± 0.05**	13.63 ± 5.00††

^∗^*P < 0.05, ^∗∗^*P* < 0.01, vs. NC; †*P* < 0.05, ††*P* < 0.01, vs. MC*.

### Changes in the Level of Fecal SCFAs During GQD Treatment

**Figure 4:**
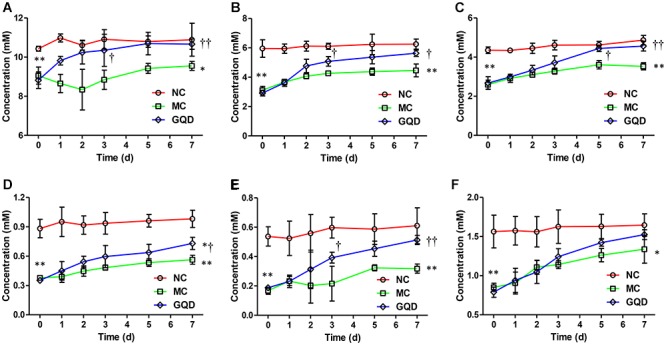
Concentrations of SCFAs in feces in each group during the treatment. Results indicate that GQD treatment could increase the levels of SCFAs. **(A)** Acetic acid. **(B)** Propionic acid. **(C)** Butyric acid. **(D)** *iso*-Butyric acid. **(E)** Valeric acid. **(F)** *iso*-Valeric acid. ^∗^*P* < 0.05, ^∗∗^*P* < 0.01, vs. NC; †*P* < 0.05, ††*P* < 0.01, vs. MC.

Six SCFAs in feces were determined via GC-MS (Supplementary Figure 4), and their concentrations were calculated through calibration curves (Supplementary Table 2). GQD treatment significantly increased the levels of fecal SCFAs in diarrheal piglets (*P* < 0.05). Acetic acid, propionic acid, and butyric acid featured high concentrations and returned to normal levels after GQD treatment (Figure 4). Moreover, over the course of the treatment, PCA revealed that the fecal SCFAs in the NC group were separated from the MC group. The fecal SCFAs in GQD group diverged from those of the MC group to resemble those of the NC group (Supplementary Figure 5). This finding is consistent with the observation of the structural modulation of gut microbiota.

### Alleviation of Mucosal Pro-inflammatory Responses in the Colon

**Figure 5:**
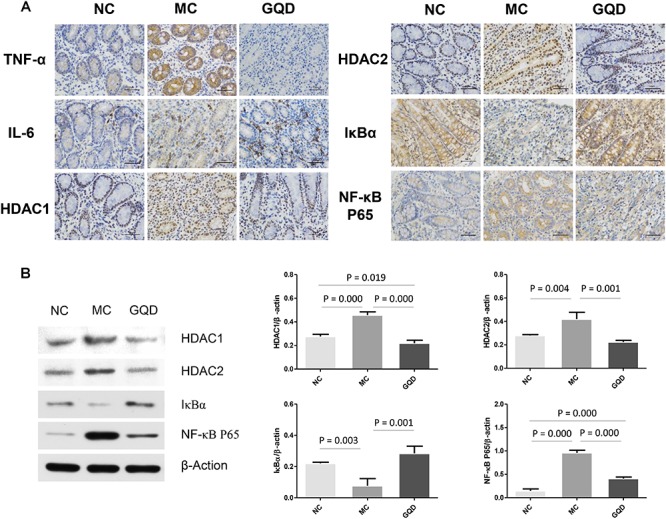
Expression of mucosal pro-inflammatory responses in piglet colon. Results suggest that SCFAs may attenuate the inflammation by inhibiting HDAC and NF-κB pathway. **(A)** Expressions of TNF-α, IL-6, HDAC1, HDAC2, IκBα, and NF-κB P65 determined by immunohistochemistry. **(B)** Western blot analysis for HDAC1, HDAC2, IκBα, and NF-κB P65.

The immunohistochemistry assessment revealed that the expressions of TNF-α, IL-6, HDAC1, and HDAC2 were heightened in the MC group and attenuated in the GQD group. Compared to the NC group, the expressions of IκBα and NF-κB P65 in the MC group were decreased and increased, respectively. However, GQD reversed these changes (Figure 5A). The ELISA test showed that the levels of TNF-α and IL-6, and HDAC and NF-κB P65 binding activities were remarkably reduced in the GQD group compared to the MC group (*P* < 0.01; Table 4). Additionally, western blot analysis revealed that the expressions of HDAC1 and HDAC2 were significantly alleviated in the GQD group compared with the MC group. The changes in the levels of IκBα and NF-κB P65 in the GQD group were increased and decreased, respectively, compared with the MC group (*P* < 0.05), which is similar to the immunochemistry assessment (Figure 5B).

**Table 4:** ELISA test of TNF-α, IL-6, and HDAC and NF-κB P65 binding activities in colonic mucosa after GQD treatment.

	NC	MC	GQD
TNF-α (pg/g)	29.08 ± 3.19	86.39 ± 9.91**	35.29 ± 7.80††
IL-6 (pg/g)	7.03 ± 1.18	12.57 ± 2.89**	7.40 ± 0.95††
HDAC activity (ng/min/mg protein)	0.045 ± 0.011	0.084 ± 0.012**	0.047 ± 0.010††
NF-κB P65 binding activity (OD_450__nm_/mg protein)	0.274 ± 0.068	0.497 ± 0.108**	0.326 ± 0.077††

^∗^*P < 0.05, ^∗∗^*P* < 0.01, vs. NC; †*P* < 0.05, ††*P* < 0.01, vs. MC*.

## Discussion

The present study conducted an *in vivo* assessment of the effects of GQD on piglets with bacterial diarrhea. Our results demonstrated a significant effect of GQD on diarrhea, evinced by a reduction in watery stool, fecal water content, colonic mucosal injury, and histological score of GQD-treated piglets. These findings agree with clinical observations (Ye et al., 2017).

As reflected by the Chao and Shannon indexes, we observed that diarrhea reduces the alpha diversity of the gut microbial community. However, the alpha diversity improved after GQD treatment, suggesting that GQD reverses the diarrhea-induced reduction in the species richness of gut microbiota. The PCA and PCoA analyses revealed significant distances between the GQD and MC groups, as well as an increasingly proximate distance between the GQD and NC groups, indicating that gut microbiota undergo structural changes with GQD treatment. These findings are consistent with previous observations that GQD improves the beta diversity of gut microbial composition (Xu J. et al., 2015). However, changes in the gut microbial composition were also observed in the NC group over the course of experiment. These results may be the product of early gut colonizers in piglets; this establishment of microbial composition and ecological succession of intestinal microbiota helps the piglets to adapt to growth, develop the immune system, and maintain long-lasting health (Dou et al., 2017; Guevarra et al., 2019). Further investigation of microbial species revealed a GQD-induced decrease and increase in the relative abundances of *Proteobacteria* and *Bacteroidetes*, respectively. In agreement with previous studies, we observed that diarrhea prompts an increase of *Proteobacteria* and thereby diminishes both the function of intestinal epithelial cells and colonization of commensal bacteria, such as *Bacteroidetes* (David et al., 2015). These results indicate that GQD modulates the composition of gut microbiota during the treatment of diarrhea.

Moreover, after treatment with GQD, we observed an increase in the relative abundances of bacteria, including *Akkermansia, Bacteroides, Butyricimonas, Clostridium, Ruminococcus*, and *Phascolarctobacterium* (*P* < 0.05). These bacteria have been identified as important commensal bacteria capable of fermenting fiber, degrading oligosaccharides, producing SCFAs, and preventing diarrhea (Kalmokoff et al., 2013; Zhang et al., 2015, 2018; Takahashi et al., 2016). By promoting these bacteria, GQD treatment may increase the levels of SCFAs. Indeed, we found that the concentrations of SCFAs in diarrheal piglets increased after GQD treatment. Interestingly, GQD treatment significantly increased the level of *A. muciniphila*, a key symbiont that degrades mucin, produces propionic acid, stimulates goblet-cell differentiation, and maintains the integrity of the mucosal barrier in the intestine (Hänninen et al., 2018). As shown in Figure 1A, diarrhea induced a decreased quantity of goblet cells, damaged the mucosal barrier, and increased gut permeability. GQD treatment reversed these changes, suggesting that GQD modulates the gut microbiota, increases SCFA levels, repairs intestinal mucosa, ameliorates permeability, and alleviates diarrhea.

**Figure 6:**
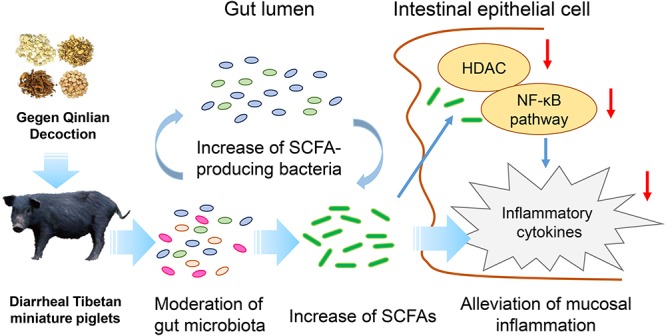
Possible mechanism underlining the GQD treats diarrhea in piglets.

Furthermore, the increase in SCFA levels may contribute to the attenuation of mucosal pro-inflammatory responses. Our analyses showed that GQD treatment decreased the levels of TNF-α and IL-6, indicating a significant GQD-induced anti-inflammatory effect. To investigate a mechanism by which SCFA, rather than GQD, attenuates mucosal inflammation, we explored the activity of its key target, HDAC (Chang et al., 2014; Park et al., 2015). The activity of HDAC was found to have significantly decreased in the group treated with GQD. These findings support previous observations that SCFAs ameliorate colonic mucosal inflammation by inhibiting HDAC (Zimmerman et al., 2012; Park et al., 2015). Moreover, GQD treatment stabilized the expression of IκBα and attenuated the level of NF-κB P65. These results are consistent with those of previous investigations: SCFAs mitigate pro-inflammatory responses by inhibiting the NF-κB pathway and may stabilize the IκBα level and suppress the NF-κB P65 binding activity (Segain et al., 2000; Liu et al., 2012; Lee et al., 2017). However, the mechanism whereby HDAC mediates the inhibitory effect on the NF-κB pathway is not clear. NF-κB is reportedly a central mediator of immune and inflammatory responses, and its activation enhances the expressions of pro-inflammatory genes (Kanarek and Ben-Neriah, 2012). However, the activation of NF-κB is regulated by the binding of inhibitory molecules, such as IκB proteins, while the degradation of the NF-κB/IκB complex is regulated by proteasomes. The activity of proteasomes could be suppressed through the inhibition of HDAC (Yin et al., 2001; Kanarek and Ben-Neriah, 2012). The observations gleaned from the present study inform our speculation that SCFAs attenuate mucosal pro-inflammatory responses by inhibiting the activity of HDAC and proteasomes, thus stabilizing the level of IκBα and preventing the activation of the NF-κB pathway (Yin et al., 2001; Place et al., 2005). Collectively, our investigation suggests that GQD treats diarrhea in piglets maybe involved in modulating gut microbiota and increasing SCFA levels (Figure 6).

The ingredients analysis demonstrated that GQD is primarily composed of antibacterial agents (Liu et al., 2018), which indicates that GQD attenuates diarrheal symptoms by promoting the destruction of pathogenic microorganisms. Recently, a clinical study that treated patients with type 2 diabetes with GQD for 12 weeks found that GQD features hypoglycemic efficacy (Xu J. et al., 2015). However, this intervention differs from the short-term use of GQD for diarrhea; its long-term usage may suppress commensal bacteria and thereby impact the balance of intestinal microbial ecology. Research on how GQD modulates the structure of intestinal microbiota is therefore necessary to inform its therapeutic application and prevent complications in its misuse.

## Conclusion

The present study first analyzed the composition of gut microbiota in diarrheal piglets receiving GQD treatment. Our results indicate that GQD increases the bacterial species richness and regulates the structure of the gut microbial community. GQD also increased the relative abundances of SCFA-producing bacteria, including *Akkermansia, Bacteroides, Clostridium, Ruminococcus*, and *Phascolarctobacterium*. Secondly, GQD treatment increased the levels of fecal SCFAs, which may closely relate to the modulation of gut microbial community. Finally, the increased SCFAs may contribute to alleviating the mucosal pro-inflammatory responses by inhibiting the HADC and NF-κB pathways in diarrheal piglets. Our findings thus indicated that microbiota play an important role in the treatment of diarrhea and that GQD administration maybe involved in promoting this effect. The underlying mechanism for this phenomenon might related to an increase in SCFAs levels and a consequent anti-inflammatory effect on intestinal mucosa.

## Ethics Statement

The animal experiments in the present study were approved by the Animal Ethics Committee of Southern Medical University. This statement was described in the manuscript.

## Author Contributions

X-MT and Q-FT conceived and designed the study. C-SL and XL performed the induction of diarrheal piglet model and drug administration. C-SL and XL performed the measurement of histology and mucosal cytokine. X-HW and ZJ contributed detection of SCFAs. F-LC contributed determination of GQD. Q-FT statistics. C-SL drafted the manuscript. All authors have read, commented on, and approved the manuscript.

## Conflict of Interest Statement

The authors declare that the research was conducted in the absence of any commercial or financial relationships that could be construed as a potential conflict of interest.
