# Supplementary material for: Gegen Qinlian Decoction Treats Diarrhea in Piglets by Modulating Gut Microbiota and Short-Chain Fatty Acids
Source: Front Microbiol. 2019 Apr 18;10:825. doi: 10.3389/fmicb.2019.00825 (PMC6482297; doi:10.3389/fmicb.2019.00825)
Supplement: Supplementary file 1 [file Data_Sheet_1.docx]

**Gegen Qinlian Decoction Treats Diarrhea in Piglets by Modulating Gut Microbiota and Short-chain Fatty Acids**

Chang-Shun Liu ^a, b, c, 1^, Xiao Liang ^a, b, c, 1^, Xiao-Han Wei ^a, b, c^, Zhen, Jin ^a, b, c^, Fei-Long Chen ^a, b, c^, Qing-Fa Tang ^a, b, c,^ *, Xiao-Mei Tan ^a, b, c,^ *

a, School of Traditional Chinese Medicine, Southern Medical University, Guangzhou 510515, PR China.

b, Guangdong Provincial Key Laboratory of Chinese Medicine Pharmaceutics, Southern Medical University, Guangzhou 510515, PR China.

c, Guangdong Provincial Engineering Laboratory of Chinese Medicine Preparation Technology, Guangzhou 510515, PR China.

*, Corresponding author: Xiao-Mei Tan (tanxm_smu@163.com), Qing-Fa Tang (tangqf96@163.com)

1, These authors contributed equally work.

**Supplementary material**

Page. 1: **Figure 1.** Representative chromatograms and UV spectrum of GQD at 275 nm determined by high-performance liquid chromatography.

Page. 2: **Figure 2.** State of feces during treatment.

Page. 3: **Figure 3.** PCA (A) and PCoA (B) analysis of gut microbial structure in piglets at 0 d.

Page. 4: **Figure 4.** Representative chromatograms SCFAs in feces determined by gas chromatography-mass spectrometer.

Page. 5: **Figure 5.** PCA analysis of SCFAs in piglet feces over the course of the GQD treatment.

Page. 6: **Table 1.** Histological score of colon tissue.

Page. 6: **Table 2.** Regression data and lower limit of quantification (LLOQ) for SCFAs.


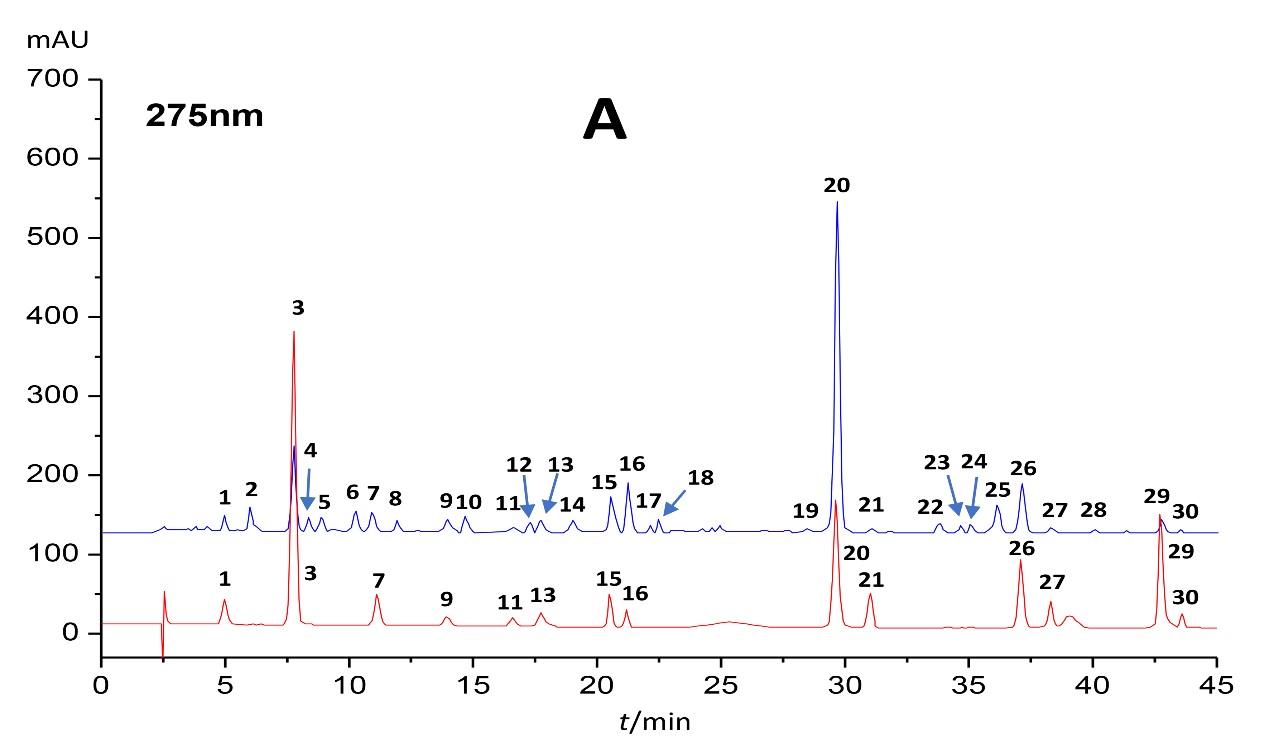


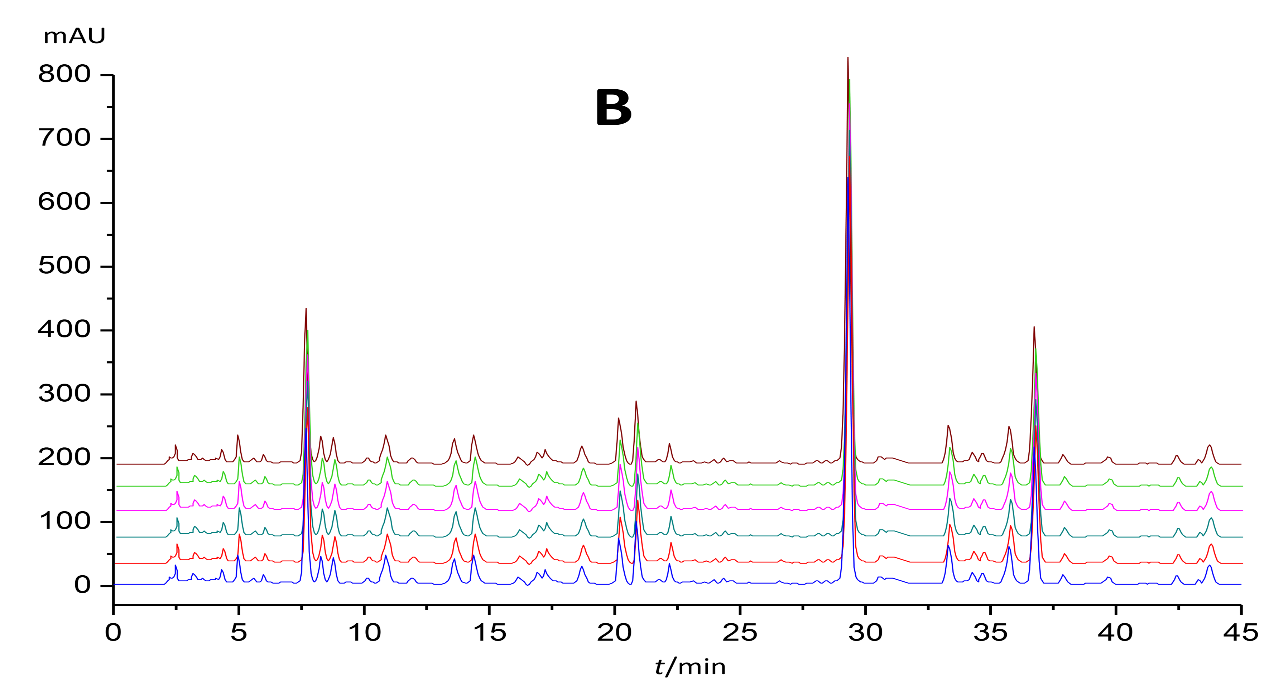


**Figure 1.** Representative chromatograms and UV spectrum of GQD at 275 nm determined by high-performance liquid chromatography. (A) 30 peaks were assigned as common peaks in the chromatograms. 14 components of GQD were identified. Peaks 2, 5, 9, 11, 21, and 27 from *Pueraria lobata* (Wild.) Ohwi were identified as puerain, 3’-hydroxypuerarin, daidzin, genistin, daidzin, and genistein; peaks 20, 26, 29, and 30 from *Scutellaria baicalensis* Georgi were identified as baicalin, wogonoside, baicalein, and wogonin; peaks 15, 17, and 18 from *Coptis chinensis* Franch. were identified as jatrorrhizine, palmatine, and berberine, and peak 13 from *Glycyrrhiza uralensis* Fisch. was identified as liquiritin. (B) The chromatograms of six groups of GQD revealed a high degree of similarity.


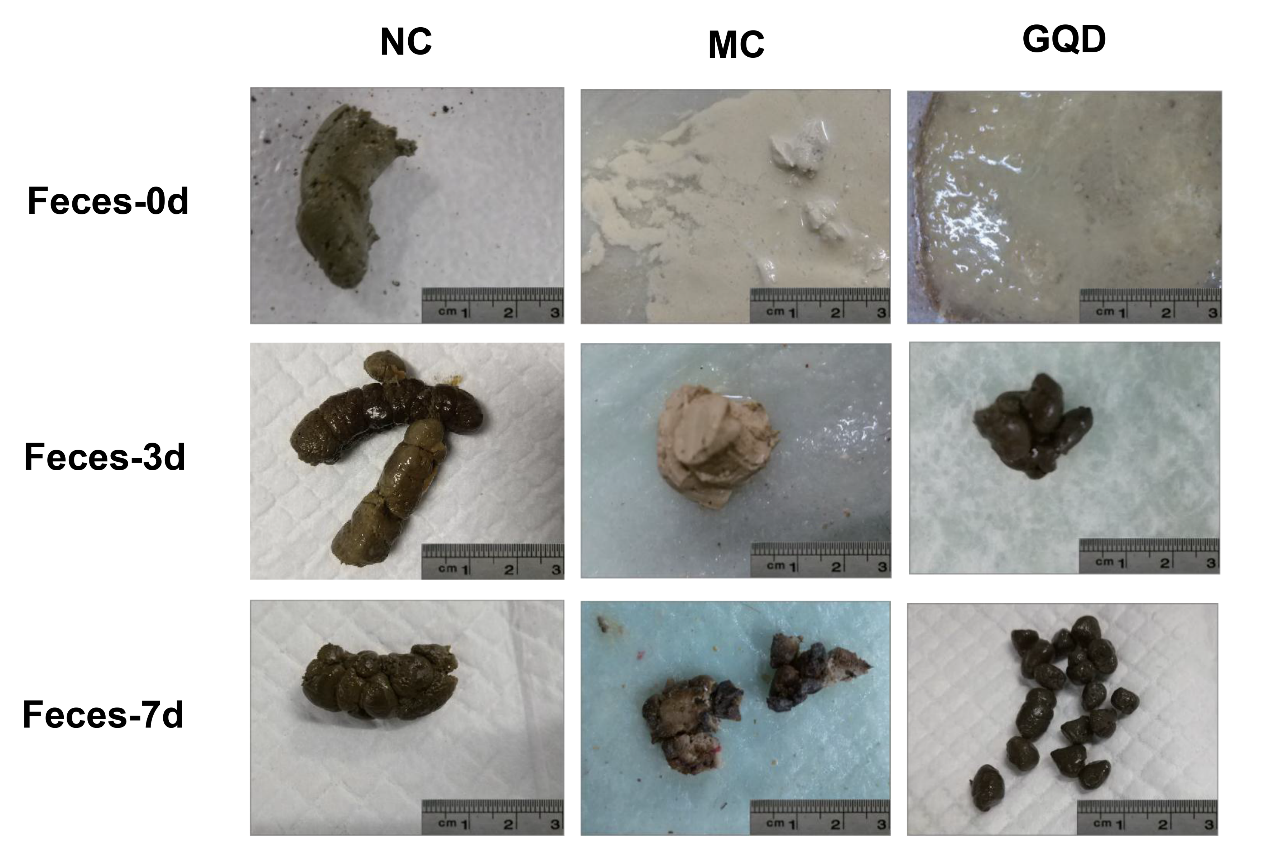


**Figure 2.** State of feces during treatment. Results showed that the diarrheal symptoms of piglets were alleviated with GQD treatment at 1 d and disappeared by 3 d.


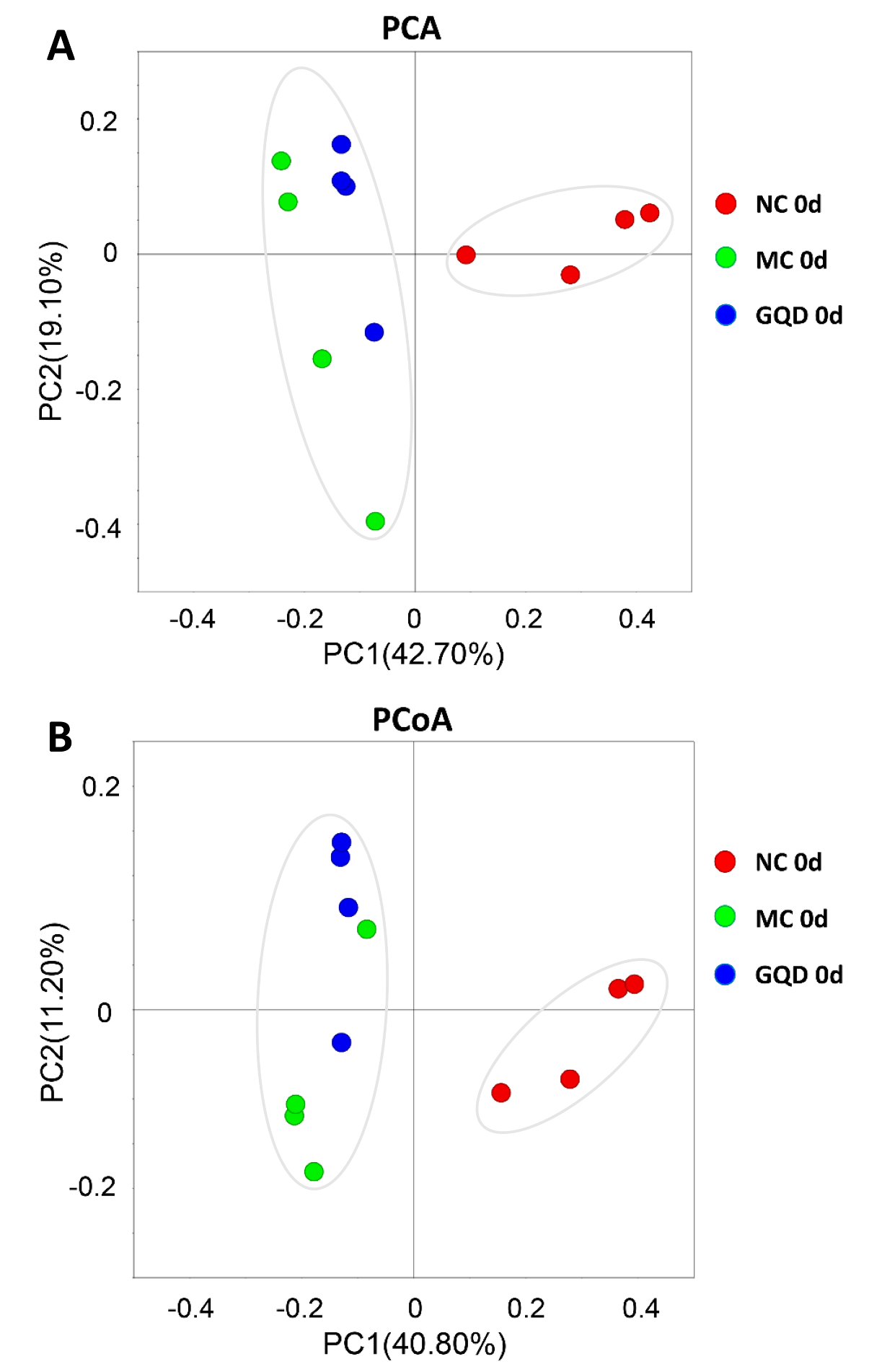


**Figure 3.** PCA (A) and PCoA (B) analysis of gut microbial structure in piglets at 0 d. Results suggest the different structure of gut microbiota between the diarrheal and healthy piglets.


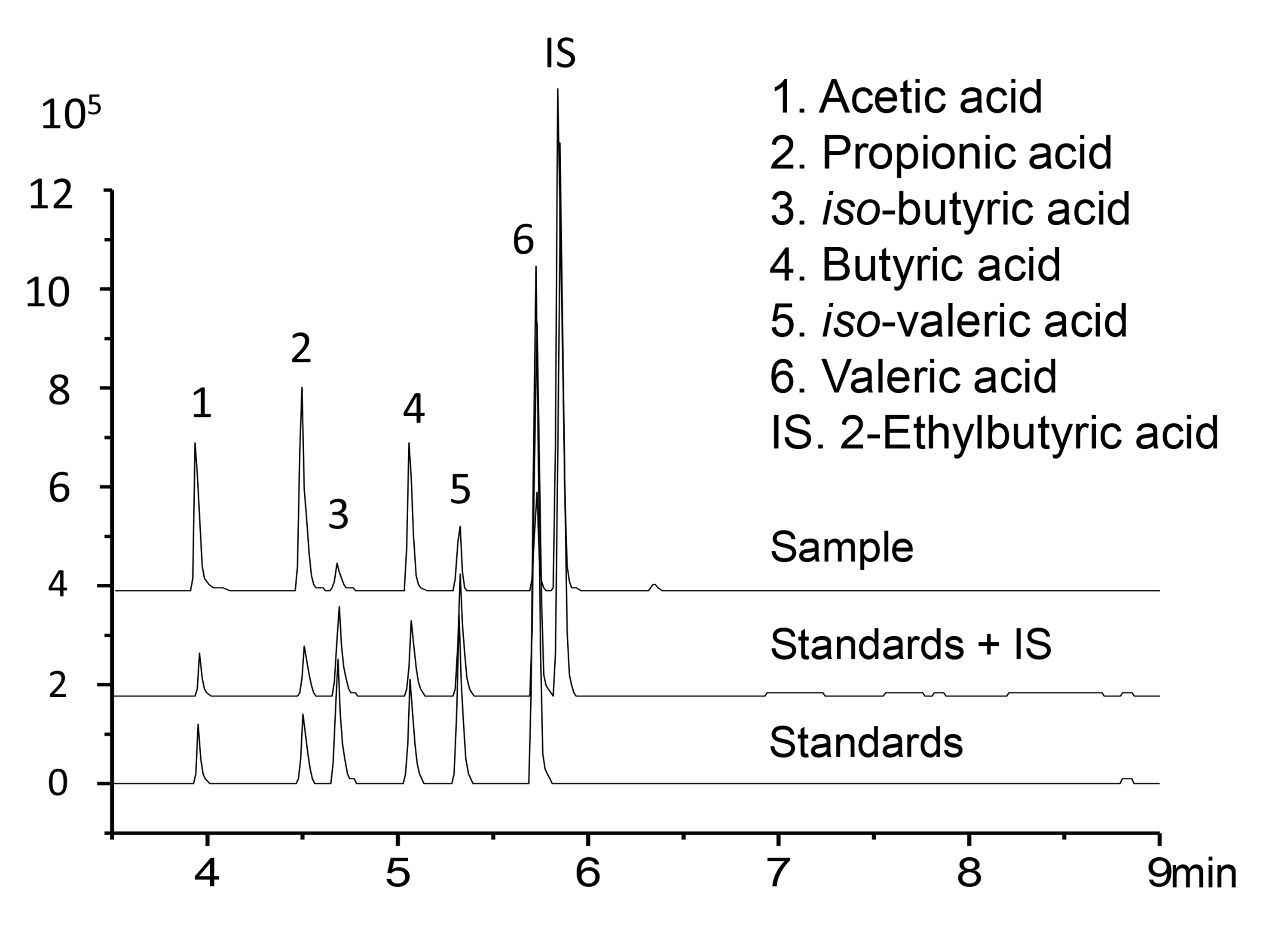


**Figure 4.** Representative chromatograms SCFAs in feces determined by GC-MS. Results showed that the six SCFAs in feces could be analyzed using a GC-MS system.


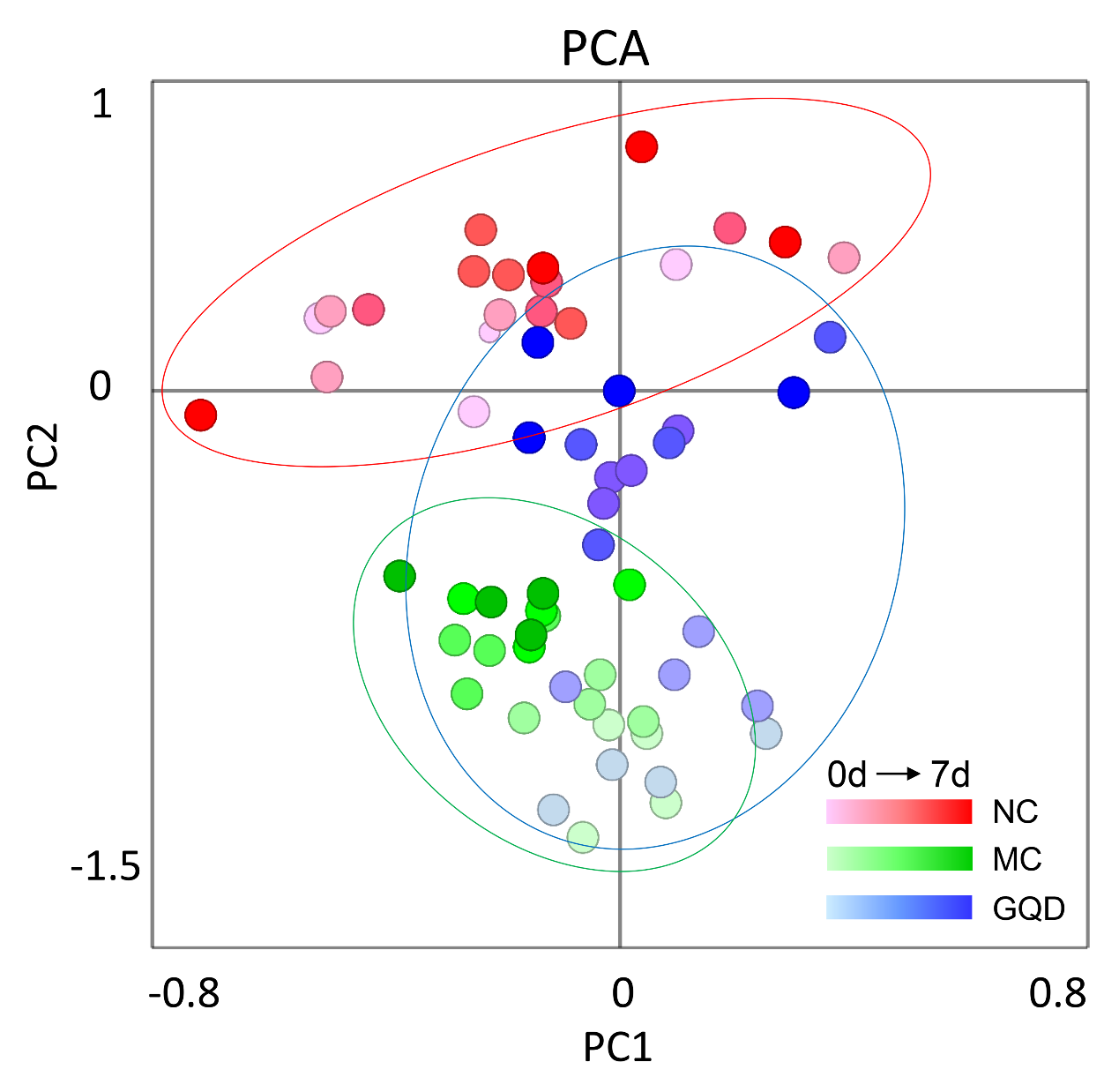


**Figure 5.** PCA analysis of SCFAs in piglet feces over the course of the GQD treatment. Results showed that the fecal SCFAs in GQD group diverged from the field of the MC group to that of NC group over the course of the treatment.

**Table 1.** Histological score of colon tissue.

| Feature score | Score | Description |
| --- | --- | --- |
| Inflammation severity | 0 | None |
|  | 1 | moderate |
|  | 2 | Severe |
| Infiltrating cells | 0 | None |
|  | 1 | Less |
|  | 2 | More |
| Goblet cells | 0 | Abundance |
|  | 1 | Less |
|  | 2 | None |
| Intercellular space | 0 | Tight |
|  | 1 | Gapped |
|  | 2 | Loose |

**Table 2.** Regression data and lower limit of quantification (LLOQ) for SCFAs.

| Compounds | Calibration curve | R^2^ | Linear range  (µg/mL) | LLOQ  (µg/mL) |
| --- | --- | --- | --- | --- |
| Acetic acid | y = 0.0012x + 0.0136 | 0.9952 | 40-500 | 40 |
| Propionic acid | y = 0.0009x + 0.0206 | 0.9932 | 40-500 | 40 |
| *iso*-butyric acid | y = 0.0022x + 0.0107 | 0.9902 | 10-100 | 10 |
| Butyric acid | y = 0.0017x + 0.0013 | 0.9917 | 20-250 | 20 |
| *iso*-valeric acid | y = 0.0024x + 0.013 | 0.9907 | 10-100 | 10 |
| Valeric acid | y = 0.0076x + 0.0248 | 0.9923 | 5-80 | 5 |
